# Phenotypic and genotypic characterization of antimicrobial resistance profiles in *Salmonella* isolated from waterfowl in 2002–2005 and 2018–2020 in Sichuan, China

**DOI:** 10.3389/fmicb.2022.987613

**Published:** 2022-10-06

**Authors:** Ying Guan, Yanwan Li, Jin Li, Zhishuang Yang, Dekang Zhu, Renyong Jia, Mafeng Liu, Mingshu Wang, Shun Chen, Qiao Yang, Ying Wu, Shaqiu Zhang, Qun Gao, Xumin Ou, Sai Mao, Juan Huang, Di Sun, Bin Tian, Anchun Cheng, Xinxin Zhao

**Affiliations:** ^1^Institute of Preventive Veterinary Medicine, Sichuan Agricultural University, Chengdu, Sichuan, China; ^2^Research Center of Avian Diseases, College of Veterinary Medicine, Sichuan Agricultural University, Chengdu, Sichuan, China; ^3^Key Laboratory of Animal Disease and Human Health of Sichuan Province, Chengdu, Sichuan, China

**Keywords:** *Salmonella*, waterfowl, prevalence, serotypes, pulsed-field gel electrophoresis, antimicrobial resistance

## Abstract

*Salmonella enterica* is a widespread foodborne pathogen with concerning antimicrobial resistance (AMR). Waterfowl are a major source of *Salmonella* transmission, but there are few systematic studies on *Salmonella* prevalence in waterfowl species. In this study, 126 *Salmonella* isolates (65 collected in 2018–2020 and 61 collected in 2002–2005) were obtained from waterfowl samples in Sichuan, China. Their serotypes, pulsed-field gel electrophoresis (PFGE) types, and phenotypic and genotypic AMR profiles were systematically examined. The isolates were distributed in 7 serotypes, including serovars Enteritidis (46.0%), Potsdam (27.8%), Montevideo (7.9%), Cerro (6.3%), Typhimurium (4.8%), Kottbus (4.0%) and Apeyeme (3.2%). Their PFGE characteristics were diverse; all isolates were distributed in four groups (cutoff value: 60.0%) and 20 clusters (cutoff value: 80.0%). Moreover, all isolates were multidrug resistant, and high rates of AMR to lincomycin (100.0%), rifampicin (100.0%), sulfadiazine (93.7%), erythromycin (89.7%), ciprofloxacin (81.0%), and gentamicin (75.4%) were observed. Finally, 49 isolates were subjected to whole-genome sequencing, and a wide variety of AMR genes were found, including multiple efflux pump genes and specific resistance genes. Interestingly, the *tet*(A)/*tet*(B) and *catII* resistance genes were detected in only isolates obtained in the first collection period, while the *gyrA* (S83F, D87N and D87G) and *gyrB* (E466D) mutations were detected at higher frequencies in the isolates obtained in the second collection period, supporting the findings that isolates from different periods exhibited different patterns of resistance to tetracycline, chloramphenicol and nalidixic acid. In addition, various incompatible plasmid replicon fragments were detected, including Col440I, Col440II, IncFIB, IncFII, IncX1, IncX9, IncI1-I and IncI2, which may contribute to the horizontal transmission of AMR genes and provide competitive advantages. In summary, we demonstrated that the *Salmonella* isolates prevalent in Sichuan waterfowl farms exhibited diverse serotypes, multiple AMR phenotypes and genotypes, and AMR changes over time, indicating their potential risks to public health.

## Introduction

*Salmonella enterica* is a facultative anaerobic gram-negative bacterium with more than 2,600 serotypes and an important zoonotic pathogen worldwide (Grimont and Weill, 2007). Human consumption of *Salmonella*-contaminated products can cause diarrhea, intestinal inflammation, and even bacteremia (Fearnley et al., 2011; Chousalkar et al., 2018; Tack et al., 2019), adding to the global burden of disease. *Salmonella* was previously reported to have caused approximately 22.2% (12,769 cases) of foodborne illness cases in China between 1994 and 2005 (Wang et al., 2007), and one of the sources of illness was waterfowl. China is the largest producer and consumer of waterfowl, such as ducks and geese, and related products (Wang et al., 2017). Recent studies have shown that waterfowl are an important source of *Salmonella* (Wang et al., 2020a; Kim et al., 2021) and often transmit *Salmonella* due to open-yard feeding (Murray et al., 2021); however, relevant systematic studies on *Salmonella* prevalence in these species have rarely been reported.

Because of diverse *Salmonella* serotypes and possible monophasic variation, simple slide agglutination assays to detect rare serotypes are often labor intensive and time consuming and have a risk of misidentification (Uelze et al., 2020). Sequence-based serotyping approaches, such as the *Salmonella in silico* typing resource (SISTR), can be used as a complementary method; this method was reported to have an accuracy of up to 94% (Yoshida et al., 2016). The *in silicon* method allows the detection of antigen genes carried by an isolate, while the slide agglutination method allows the detection of antigens expressed by an isolate (Yachison et al., 2017). A combination of these two methods can yield more accurate results. Pulsed-field gel electrophoresis (PFGE) has emerged as a method for analyzing large molecules of DNA (Sharma-Kuinkel et al., 2016) and has been widely used in molecular epidemiological investigations of foodborne pathogens (e.g., *Escherichia coli*, *Salmonella enterica* and *Listeria monocytogenes*; Favier et al., 2013; Li et al., 2020a). PFGE results reflect the genetic relationships among different isolates, allowing the rapid monitoring, tracking and tracing of bacterial infections.

In recent decades, antimicrobial agents have been used frequently in animal husbandry not only to treat and control *Salmonella* and other pathogens but also as prophylactic measures and growth-promotors. The abuse and misuse of antimicrobials has led to antimicrobial resistance (AMR), which is still increasing (Palma et al., 2020). To address this problem, use of antimicrobials as a growth-promoting factors has been banned in the European Union since 2006 (Castanon, 2007). China is one of the world’s largest producers and consumers of antimicrobials, with 162,000 tons of antimicrobials used in 2013, 52% of which were for veterinary use (Zhang et al., 2015). Over the past two decades, China’s restrictive policies on veterinary antimicrobials have changed substantially and become increasingly stringent (Yinqi et al., 2019). Consequently, the prevalence of colistin-resistant *Escherichia coli* in pigs and chickens decreased dramatically from 2015 to 2018 due to the withdrawal of colistin as an animal growth promoter in China (Wang et al., 2020b), highlighting the impact of addressing AMR. Nevertheless, there are still numerous reports showing that the *Salmonella* prevalence in poultry, pigs and eggs in China had exhibited increasing resistance to multiple antimicrobials (Yang et al., 2019; Li et al., 2020b, 2021; Xu et al., 2021). Thus, it is of interest and importance to perform continuous AMR monitoring and to investigate changes in AMR over time, which is crucial in identifying the mechanisms involved and providing guidance on rational treatment strategies. Characterization of *Salmonella* resistance is mainly performed by determining phenotypes based on antimicrobial susceptibility testing (AST; CLSI, 2020) and genotypes based on whole-genome sequencing (WGS; Schwan et al., 2021; Yan et al., 2021; Medina-Santana et al., 2022). Phenotypes usually correspond to the external expression of a single gene but may also be the result of synergistic effects of multiple genes (Morales et al., 2005). Therefore, both phenotypic and genotypic testing are necessary for pathogen surveillance and diagnosis, and using them together can lead to more accurate judgments.

In this study, 126 *Salmonella* isolates (including 65 collected in 2018–2020 and 61 collected in 2002–2005) obtained from waterfowl samples in Sichuan, China, were subjected to serotyping, PFGE molecular typing, AST, and WGS to investigate their prevalence and AMR profiles.

## Materials and methods

### Sample collection and bacterial isolation

All samples were collected from waterfowl farms with animal deaths, diarrhea or declining egg production. One hundred and 21 duckling organ samples were collected from Pujiang and Xinjin farms in 2002–2005, and 150 samples, including 70 duck fecal samples, 43 duck cloacal samples and 37 goose egg samples, were collected from five farms in Dayi, Chongzhou, Jintang, Mianyang and Pengzhou of Sichuan Province, China, in 2018–2020. All samples were subjected to isolation according to a standard protocol described previously (Andrews et al., 2022). In brief, for goose eggs and cloacal samples, samples were collected with sterile swabs and diluted in 1 ml of phosphate-buffered saline (PBS). Then, 100 μl of this solution was added to 10 ml of buffered peptone pre-enrichment solution, followed by incubation at 37°C for 24 h. For fecal and organ samples, 1 g of each was weighed, ground and added to 10 ml of buffered peptone pre-enrichment solution, followed by incubation at 37°C for 24 h. Subsequently, 1 ml of pre-enrichment solution was added to 10 ml of *Salmonella*-specific selenite cystine (SC) enrichment solution, followed by incubation at 37°C for 24 h, and then 100 μl of the solution was applied to plates with xylose lysine deoxycholate agar (XLD) medium. Putative black colonies on XLD medium were selected and subjected to polymerase chain reaction (PCR) identification by using *Salmonella*-specific primers hut-F/R (hut-F: atgttgtcctgcccctggtaagaga, hut-R: actggcgttatccctttctctg) to confirm (Alzwghaibi et al., 2018). All identified isolates were stored in 15% (*v*/*v*) glycerol at −80°C.

### Serotyping and pulsed-field gel electrophoresis typing

Serotyping of the isolates was carried out by slide agglutination of flagellar antigen (H) and somatic antigen (O) with a *Salmonella* Diagnostic Serum Kit (Tianrun Biopharmaceuticals, Ningbo, China) according to the manufacturer’s instructions (Lee et al., 2015). Some nonagglutinable isolates were further determined by the SISTR v1.1.1 using WGS data (Yoshida et al., 2016).

The genetic relationships among the isolates were determined by the PFGE method according to the PulseNet protocol (CDC, 2017). *Xba*I (New England Biolabs, Ipswich, MA, USA) was used as the restriction enzyme. Clustering analysis was performed by Bionumerics v7.6 (Applied Maths NV, Sint-Martens-Latem, Belgium) using the unweighted pair-group average method with band-matching settings of 1.0% optimization and 1.5% position tolerance (Tian et al., 2021). *Salmonella enterica* serovar Braenderup H9812 was included for quality control.

### Antimicrobial susceptibility testing

The susceptibility of the isolates to ten classes of 20 antimicrobials was determined by the Kirby-Bauer disk diffusion method (Bauer et al., 1966). The categories (susceptible, intermediate resistance or resistance) were interpreted according to the Clinical and Laboratory Standards Institute (CLSI) guidelines (CLSI, 2020). The antimicrobial agents employed were as follows: tetracycline (TET, 30 μg), aztreonam (ATM, 30 μg), ampicillin (AMP, 10 μg), trimethoprim/sulfamethoxazole (SXT, 1.25/23.75 μg, respectively), nalidixic acid (NAL, 30 μg), gentamycin (GEN, 10 μg), amoxicillin (AML, 25 μg), chloramphenicol (CHL, 30 μg), polymyxin B (PB, 300 μg), streptomycin (STR, 10 μg), trimethoprim (W, 5 μg), sulfadiazine (SUL, 100 μg), ciprofloxacin (CIP, 5 μg), ceftiofur (EFT, 30 μg), cefepime (FEP, 30 μg), imipenem (IPM, 10 μg), lincomycin (MY, 2 μg), florfenicol (FFC, 30 μg), erythromycin (E, 15 μg), and rifampin (RD, 5 μg). The *Escherichia coli* reference strain ATCC 25922 was used for quality control. Two-way ANOVA and the Pearson chi-square test were used to determine the difference in the overall AMR rates of *Salmonella* and the difference in the AMR rates to a particular antimicrobial agent between the two periods.

### Whole-genome sequencing

Based on the results of PFGE typing and AST, 49 isolates with less than 80% homology, different antimicrobial phenotypes or nonagglutinable phenotypes were subjected to WGS. Their genomic DNA was extracted by using a bacterial genomic DNA extraction kit (TIANGEN Biotechnology Co., Ltd., Beijing, China). The genomic DNA was sent to the Beijing Genomics Institute (BGI Co., Ltd., Shenzhen, China) for frame sequencing (Illumina HiSeq 2000) and splicing (SPAdes v3.14.0). The sequencing results were analyzed using the Comprehensive Antimicrobial Resistance Database (CARD v3.2.4; Alcock et al., 2020) to annotate the AMR genes of the isolates and to analyze the relationships between genotypes and phenotypes. Incompatible fragments of plasmids were predicted using PlasmidFinder v2.0.1 with a similarity cutoff value of 95% (Camacho et al., 2009; Carattoli et al., 2014). Average nucleotide identity (ANI) levels were calculated by using CJ Bioscience’s online calculator (Yoon et al., 2017).

## Results

### Isolation and serotyping of *Salmonella* isolates

Sixty-five *Salmonella* isolates were obtained from 150 samples collected in 2018–2020, with an isolation rate of 43.3% (65/150); additionally, 61 isolates were obtained from 121 organ samples collected in 2002–2005, with an isolation rate of 50.4% (61/121). Serotyping with antiserum found that 108 of the 126 *Salmonella* isolates were agglutinable and 18 isolates were untypable. The uncertain serotypes were further determined by using an *in silico* typing method. A total of seven serotypes were detected and distributed in six serogroups: serovar Typhimurium in group B; serovar Montevideo and Potsdam in group C1; serovar Kottbus in group C2; serovar Apeyeme in group C3; serovar Enteritidis in group D1; and serovar Cerro in group K (Table 1). The serotype distribution of these isolates was as follows: 46.0% (58/126) were serovar Enteritidis; 27.8% (35/126) were serovar Potsdam; 7.9% (10/126) were serovar Montevideo; 6.3% (8/126) were serovar Cerro; 4.8% (6/126) were serovar Typhimurium; 4.0% (5/126) were serovar Kottbus; and 3.2% (4/126) were serovar Apeyeme (Table 1).

**Table 1:** Isolation and serotyping of *Salmonella* from waterfowl samples.

Farm	Collection time	Source	Number of samples	Serotype (serogroup)							Number of isolates
				Enteritidis (D1)	Potsdam (C1)	Montevideo (C1)	Cerro (K)	Typhimurium (B)	Kottbus (C2)	Apeyeme (C3)	
Dayi	2020	Duck cloaca	6	0	0	0	0	3	0	0	3
Chongzhou	2019	Duckling cloaca	9	0	1	2	0	0	0	2	5
Jintang	2018	Duck feces	70	0	0	0	0	0	2	0	2
Mianyang	2019	Duckling cloaca	28	0	0	8	8	0	3	2	21
Pengzhou	2020	Goose eggs	37	0	34	0	0	0	0	0	34
Pujiang	2002–2004	Duckling spleen and liver	69	31	0	0	0	2	0	0	33
Xinjin	2003–2005	Duckling spleen and liver	52	27	0	0	0	1	0	0	28
Total			271	58	35	10	8	6	5	4	126

### Molecular typing of *Salmonella* isolates by pulsed-field gel electrophoresis

**Figure 1:**
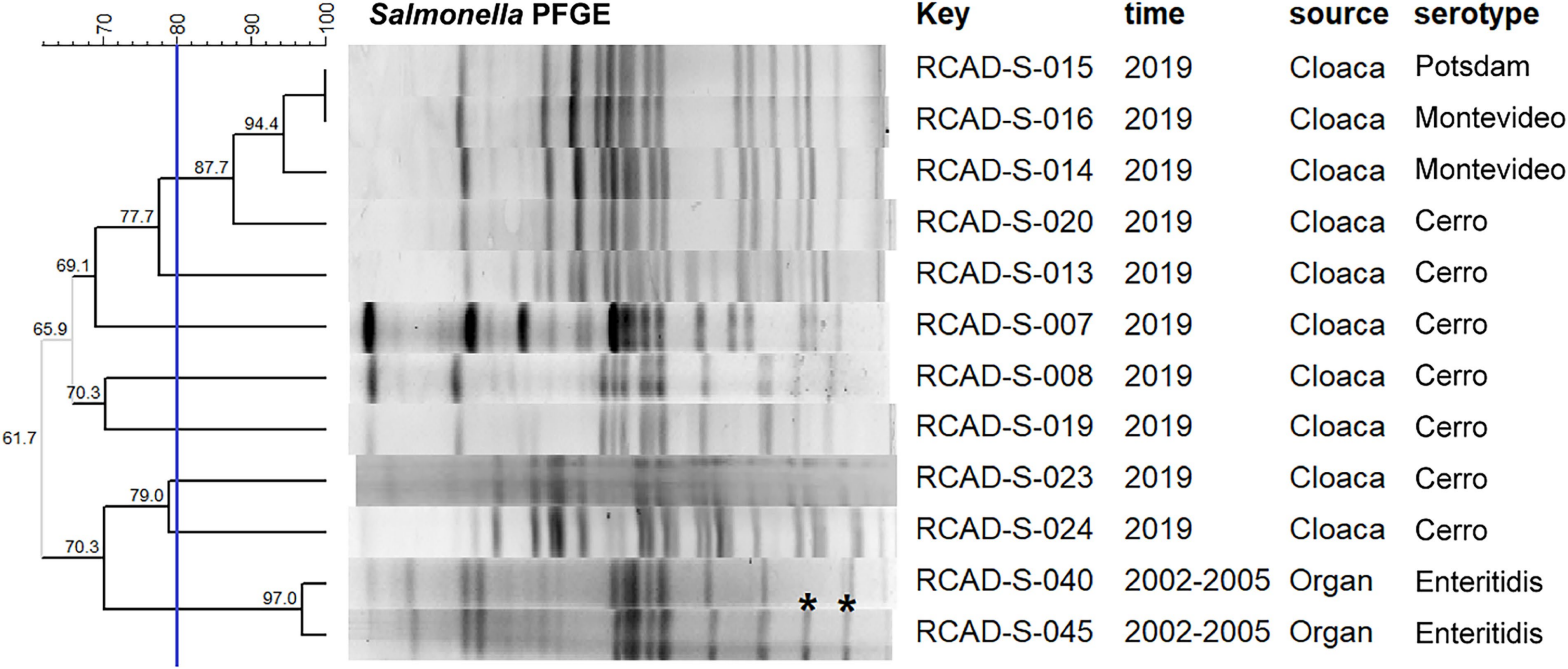
Dendrogram of the PFGE pulsotypes for 12 representative isolates. The blue line indicates the 80% cutoff value. The “key” column represents the different isolates; RCAD, Research Center of Avian Diseases. Asterisks indicate differences between adjacent bands.

PFGE typing of the 126 isolates resulted in diverse band characteristics, with all isolates classified into four groups (cutoff value: 60.0%), designated A, B, C and D, containing 58, 38, 9 and 21 isolates, respectively; moreover, the pulsotypes were further subdivided into 20 clusters (cutoff value: 80.0%; Supplementary Figure S1). Notably, all serovar Enteritidis isolates collected in 2002–2005 belonged to group A, with a relatively high similarity of 78.0%; all 34 isolates obtained from goose eggs in 2020 belonged to group B, while an isolate named RCAD-S-122 had only 66.7% homology with the remaining 33 isolates (Supplementary Figure S1). In contrast, the isolates from fecal and cloacal samples collected in 2018–2020 showed diversity on the PFGE dendrogram, with 21 isolates in group D, 6 isolates in group C, and 4 isolates in group B (Supplementary Figure S1). Moreover, there were no significant correlations between serotypes and pulsotypes within some isolates. For example, two (RCAD-S-023 and RCAD-S-024) of the Cerro isolates had only 61.7% PFGE homology with the remaining Cerro isolates; additionally, the serovar Potsdam isolate RCAD-S-015 and the serovar Montevideo isolate RCAD-S-016 showed identical pulsotypes with 100% PFGE homology (Figure 1). Comparison of the genome sequences with similar band characteristics found that the ANI levels between RCAD-S-015 and RCAD-S-016 were lower than those of RCAD-S-023 and RCAD-S-024 with the same serotype (98.3% versus 99.8%; Supplementary Table 1).

### Antimicrobial resistance of *Salmonella* isolates

All isolates were resistant to three or more classes of antimicrobials, suggesting multidrug resistance (MDR; Supplementary Table 2). The isolates displayed varying rates of resistance to the 20 antimicrobial agents that were tested. High resistance rates were observed for MY (100.0%), RD (100.0%), SUL (93.7%), E (89.7%), CIP (81.0%), and GEN (75.4%); however, the rates of resistance to the remaining 14 antimicrobials, namely, EFT (46.8%), TET (41.3%), AMP (24.6%), STR (20.6%), ATM (16.7%), NAL (15.1%), IPM (9.5%), AML (7.9), SXT (7.1%), CHL (7.1%), FEP (5.6%), W (4.0%), FFC (3.2%) and PB (1.6%), were less than 50%, indicating that these isolates were generally more resistant to conventional antimicrobials (Table 2).

**Table 2:** AST of the 126 *Salmonella* isolates.

Antimicrobial	Serotype							Resistance		
	Enteritidis(*n* = 58)	Potsdam(*n* = 35)	Montevideo (*n* = 10)	Cerro (*n* = 8)	Typhimurium (*n* = 6)	Kottbus (*n* = 5)	Apeyeme (*n* = 4)	2002–2005 (%, *n* = 61)^a^	2018–2020 (%, *n* = 65)^a^	Total (%, *n* = 126)
Aminoglycoside										
GEN	42	33	3	6	4	4	3	43 (70.5)	52 (80.0)	95 (75.4)
STR	10	4	2	3	2	2	3	10 (16.4)	16 (24.6)	26 (20.6)
Beta-lactams										
AML	4	1	1	3	0	1	0	4 (6.6)	6 (9.2)	10 (7.9)
EFT	14	24	6	7	2	3	3	14 (23.0)^b^	45 (69.2)^b^	59 (46.8)
AMP	20	0	4	6	0	1	0	20 (32.8)	11 (16.9)	31 (24.6)
ATM	10	5	1	2	0	2	1	10 (16.4)	11 (16.9)	21 (16.7)
IPM	9	0	0	0	0	1	2	9 (14.8)	3 (4.6)	12 (9.5)
FEP	5	0	0	0	0	1	1	5 (8.2)	2 (3.1)	7 (5.6)
Amphenicol										
CHL	4	0	1	2	0	2	0	4 (6.6)	5 (7.7)	9 (7.1)
FFC	0	1	1	1	0	1	0	0 (0)	4 (6.2)	4 (3.2)
Sulfonamides/Trimethoprim										
SUL	58	34	3	8	6	5	4	61 (100.0)	57 (87.7)	118 (93.7)
W	0	1	0	3	0	1	0	0 (0)	5 (7.7)	5 (4.0)
SXT	2	3	0	3	0	1	0	2 (3.3)	7 (10.8)	9 (7.1)
Polymyxin										
PB	0	1	0	0	1	0	0	0 (0)	2 (3.1)	2 (1.6)
Quinolone										
NAL	4	0	9	5	0	1	0	4 (6.6)^c^	15 (23.1)^c^	19 (15.1)
CIP	50	24	10	6	6	2	4	53 (86.9)	49 (75.4)	102 (81.0)
Tetracycline										
TET	44	0	2	3	1	1	1	45 (73.8)^b^	7 (10.8)^b^	52 (41.3)
Lincosamides										
MY	58	35	10	8	6	5	4	61 (100.0)	65 (100.0)	126 (100.0)
Macrolides										
E	56	34	6	5	6	3	3	59 (96.7)	54 (83.1)	113 (89.7)
Rifamycin										
RD	58	35	10	8	6	5	4	61 (100.0)	65 (100.0)	126 (100.0)

Numbers represent the isolates that were resistant to the corresponding antimicrobial agents for different serotypes or different times. SUL, sulfadiazine; CIP, ciprofloxacin; GEN, gentamycin; EFT, ceftiofur; TET, tetracycline; AMP, ampicillin; STR, streptomycin; ATM, aztreonam; NAL, nalidixic acid; IPM, imipenem; SXT, trimethoprim/sulfamethoxazole; AML, amoxicillin; CHL, chloramphenicol; FEP, cefepime; W, trimethoprim; FFC, florfenicol; and PB, polymyxin B.

a Indicates the overall difference in AMR rates between the two periods (two-way ANOVA test, *p* < 0.0001).

b Indicates a significant difference in AMR rates of specific antimicrobial agents between the two periods (Chi-square test, *p* < 0.0001).

c Indicates a significant difference in AMR rates of specific antimicrobial agents between the two periods (Chi-square test, *p* < 0.005).

The 61 isolates from the first collection period (2002–2005) exhibited higher resistance rates to SUL, E, CIP, TET, AMP and IPM than the 65 isolates from the second collection period (2018–2020); in contrast, the isolates from the second collection period showed higher resistance rates to GEN, EFT, STR, NAL and SXT (Table 2). In particular, dramatic differences in AMR rates were observed for EFT (23.0% versus 69.2%) and TET (73.8% versus 10.8%) in isolates from both periods (Table 2). Additionally, diverse resistance phenotypes were observed between isolates with the same serotype and with high homology of pulsotypes. For example, the two serovar Enteritidis isolates RCAD-S-040 and RCAD-S-045 showed 97.0% pulsotype similarity, with only slight differences found in the low-molecular weight bands (Figure 1), but they displayed quite different resistance profiles to 7 antimicrobials (TET, AMP, STR, ATM, NAL, SXT, and CHL; Supplementary Table 2).

### Analysis of antimicrobial resistance phenotypes and genotypes

**Figure 2:**
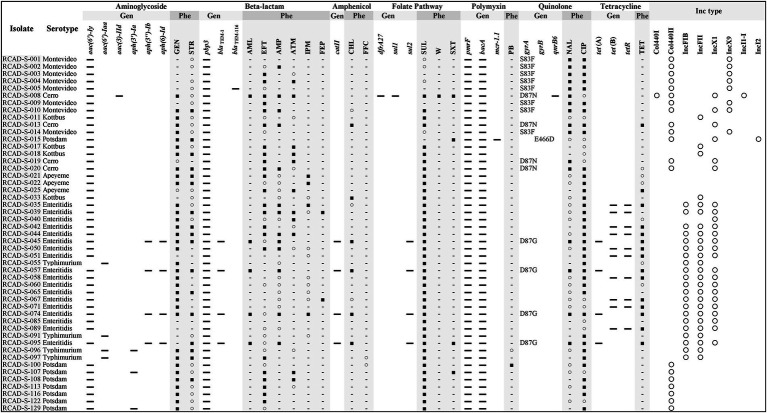
Comparison of antimicrobial resistance (AMR) profiles and plasmid replicons of the 49 *Salmonella* isolates. For the *gyrA* and *gyrB* genes, mutation sites are shown. Gen, genotype; Phe, phenotype; and Inc. type, incompatible type. ▬, Hit resistance gene; ■, resistant; ○, susceptible, increased exposure; −, susceptible, standard dosing regimen; and ○, hit plasmid replicons. SUL, sulfadiazine; CIP, ciprofloxacin; GEN, gentamycin; EFT, ceftiofur; TET, tetracycline; AMP, ampicillin; STR, streptomycin; ATM, aztreonam; NAL, nalidixic acid; IPM, imipenem; SXT, trimethoprim/sulfamethoxazole; AML, amoxicillin; CHL, chloramphenicol; FEP, cefepime; W, trimethoprim; FFC, florfenicol; and PB, polymyxin B.

Multiple specific AMR genes (*aac(6′)-Iy*, *aac(6′)-Iaa*, *aac(3)-IId*, *aph(3′)-Ia*, *aph(3″)-Ib*, *aph(6)-Id*, *bla*_TEM-1_, *bla*_TEM-116_, *catII*, *dfrA27*, *sul1*, *sul2*, *mcr-1.1*, *gyrA*, *gyrB*, *qnrB6*, *tet*(A), *tet*(B) and *tetR*) and efflux pump genes (*mdsA*, *adeF*, *golS*, *sdiA*, *acrA*, *acrB*, *marA*, *marR*, *baeR*, *baeS*, *rsmA*, *crp*, *H-NS*, *mdtK*, *mdfA*, *kpnE*, *kpnF*, *emrB*, *emrR*, *soxS*, *soxR*, *msbA*, *glpT*, *uhpT*, *EF-Tu* and *kdpE*) were detected in the overall genome or plasmid sequences of the 49 isolates (Supplementary Table 3). Analysis of AMR phenotypes and the specific AMR genes in each isolate indicated that there were specific AMR genes, including the beta-lactam inactivating enzyme gene *bla*_TEM-1_, amphenicol inactivating enzyme gene *catII* and tetracycline efflux pump genes *tet*(A) and *tet*(B), existing in the isolates with phenotypic resistance to AML, CHL and TET, respectively, whereas there were no direct correspondences to known AMR genes in isolates with phenotypic resistance to MY, RD, E, GEN, STR, EFT, ATM, SUL and CIP (Figure 2). Furthermore, in contrast to *tet* genes (which provide the TET-resistant phenotype), which were present in only the isolates obtained in the first collection period, abundant *gyrA* (S83F, D87N, and D87G) and *gyrB* (E466D) mutations (providing the NAL-resistant phenotype) were detected in the isolates obtained in the second collection period (Figure 2).

Plasmid replicon fragments with different incompatibility groups, including Col440I, Col440II, IncFIB, IncFII, IncX1, IncX9, IncI1-I, and IncI2, were detected in 46 of the 49 isolates. Most isolates contained more than one replicon fragment, such as RCAD-S-008 (Col440I, Col440II, IncX1, and IncI1-I), indicating that these isolates may harbor one or more plasmids (Figure 2). The incompatible fragments contained in isolates from different times or sources differed significantly, with isolates from the first collection period mainly containing IncFIB, IncFII, and IncX1, while isolates from the second collection period showed more diversity, except the goose egg isolates, which contained only Col440II (Figure 2). It is worth noting that although RCAD-S-040 and RCAD-S-045 share the same serotype and have high PFGE (97.0%) and ANI (99.9%) similarity (Figure 1; Supplementary Table 1), their AMR genotypes and phenotypes are significantly different, with RCAD-S-045 additionally containing IncFIB and IncFII, as well as the *gyrA* (D87G) mutation and 6 resistance genes [(*aph(3″)-Ib*, *aph(6)-Id*, *bla*_TEM-1_, *catII*, *sul2* and *tet*(A)); Figure 2].

## Discussion

The seven serotypes identified in this study showed some diversity, and the serovars Enteritidis, Montevideo, Potsdam and Typhimurium are also reported to be the major serotypes prevalent in the global poultry industry (Shah et al., 2017; Yang et al., 2020; Diaz et al., 2021). The high homology of the PFGE patterns among 58 serovar Enteritidis isolates and 34 serovar Potsdam isolates indicated that a dominant clone was prevalent locally. In contrast, the remaining isolates showed quite dissimilar PFGE patterns, suggesting that they have genotypic diversity and that various *Salmonella* clones were prevalent in different waterfowl farms in Sichuan, China. As with many epidemiological surveys, the prevalence of a particular pathogen is usually regional in nature (Yan et al., 2021). Comparisons of PFGE and serotype results revealed the following three association patterns. First, most of the isolates with the same serotypes exhibited similar PFGE band distributions and thus were grouped into the same PFGE cluster, such as serovar Enteritidis and Potsdam isolates; this indicated that their genomic arrangements were similar and that their homology was relatively high. Second, several isolates with the same serotype, such as serovar Cerro, exhibited significant heterogeneity in their PFGE patterns, with alterations in the band distributions, suggesting that these isolates may have undergone genomic rearrangement, resulting in changes in the enzymatic cutting site that did not affect their surface antigen composition. This phenomenon is supported by a previous report that 46 *Salmonella enterica* serovar Schwarzengrund isolates, with a considerable length of evolutionary time, are still of the same serotype (Yang et al., 2022). Finally, individual isolates exhibited similar pulsotypes but different serotypes; this was observed between the serovar Potsdam isolate RCAD-S-015 (6,7,14:l,v,e,n,z_15_) and the serovar Montevideo isolate RCAD-S-016 (6,7,14,[54]:g,m,s:-). Previous reports have also shown that epidemiologically unrelated isolates can be assigned to identical PFGE types (Barco et al., 2013; Shi et al., 2015), such as serovar Typhimurium (1,4,[5],12:i:1,2) versus 4,5,12:i:- (Ranieri Matthew et al., 2013) and serovar Thompson (6,7,14:k,1,5) versus 1,7:-:1,5 (Soyer et al., 2010). Since *Xba*I PFGE may not be discriminatory enough in some cases, we confirmed by WGS that the level of ANI between these two isolates was not as high as those between the same serotypes.

Owing to the excessive use of antimicrobial agents in animal husbandry over the last few decades and the horizontal spread of resistance genes, AMR in *Salmonella* has become a major concern (Foley et al., 2008). All isolates identified in this study were MDR and were broadly resistant to conventional antimicrobials, suggesting that most bacteria have acquired resistance traits under prolonged selection pressures, which is consistent with many previous findings (Yang et al., 2020; Sun et al., 2021; Wang et al., 2021). We also found variations in the resistance patterns of isolates from different periods, with the isolates obtained in the first collection period exhibiting a marked increase in resistance to EFT and newer-generation antimicrobials as well as an increase in susceptibility to TET. This trend may be attributed to the “strictest antibiotic regulation” enacted by the Chinese government in 2012, which allowed the use of veterinary antimicrobials such as EFT (Yinqi et al., 2019); increased resistance to EFT was also reported in *Salmonella* from pigs in Sichuan from 2009 (11.4%) to 2014 (53.8%; Xiuying et al., 2015). In contrast, a study from Canada showed the opposite trend, with resistance to EFT decreasing from 62 to 18% from 2004 to 2008 (Dutil et al., 2010). Variations in resistance over time suggest that the resistance phenotypes may change with the use of veterinary antimicrobials in upcoming years (Hornish and Kotarski, 2002; Sato et al., 2014). In particular, a small proportion of isolates also showed moderate resistance to human-restricted antimicrobials, such as IPM and SXT, implying possible cross-transmission between humans and animals or, more likely, the acquisition of new specific resistance genes (Wall et al., 2016).

WGS has become a reliable method for the detection of resistance genes, allowing the accurate identification of individual resistance genes in addition to the identification of single nucleotide mutations; this knowledge may even be applied to predict unknown resistance genes according to their conserved structural domains (Köser et al., 2014; Rokney et al., 2020). Specific resistance genes against different types of antimicrobials were detected in the 49 isolates. Some of the resistance genomic and phenotypic characteristics were consistent; for instance, the presence of the *catII* and *tet* genes conferred CHL and TET resistance phenotypes, respectively. However, there were substantial inconsistencies, and some isolates did not contain specific resistance genes but showed a resistance phenotype, such as serovar Potsdam isolated from goose eggs with the CIP phenotype, which may be attributed to unknown resistance mechanisms or nonspecific functions of multiple redundant efflux pump-like genes. As previously reported, CIP resistance is influenced by the coordination between multiple genes (*gyrA*, *gyrB*, *parE*, and *acrB*; O'Regan et al., 2009). Additionally, some isolates possessed specific resistance genes but showed susceptibility to the corresponding antimicrobial, which may be due to mutations or functional incompleteness of the gene; for example, *bla*_TEM-116_ in RCAD-S-005 was not observed to be resistant to beta-lactams. Eight types of incompatible plasmid replicon fragments were detected in these 49 isolates. It is reasonable to assume that the AMR differences between the representative isolates RCAD-S-040 and RCAD-S-045 stems from the different plasmids they contain, and the same situation exists for RCAD-S-014 and RCAD-S-16. Since WGS does not provide a complete map, it is not possible to determine which plasmid a specific resistance gene is located on. As plasmids play a vital role in the horizontal transfer of resistance genes, their sequences and contributions to AMR need further investigation. Overall, the analysis of resistance phenotypes and genotypes suggests that their AMR profiles may be a result of long-term stress through mechanisms such as target alteration or horizontal gene transfer by mobile genetic elements (Bakkeren et al., 2022; Cohen et al., 2022).

Since our samples were obtained from waterfowl with pathological symptoms, we obtained a higher isolation rate than those reported in other studies and consequently a higher rate of AMR (Chen et al., 2020; Han et al., 2020). The shortcomings of our study include an insufficient sample size and the lack of a wide geographical distribution. Nevertheless, our study not only complements the epidemiological surveillance data for monitoring *Salmonella* of waterfowl origin but also has practical implications for guiding the use of antimicrobial agents in waterfowl in this region.

## Data availability statement

The datasets presented in this study can be found in online repositories. The names of the repository/repositories and accession number(s) can be found at: NCBI: PRJNA857101.

## Author contributions

XZ and AC conceived the study and edited the manuscript. YG and YL performed most of the research and drafted the manuscript. JL, DZ, RJ, ML, MW, and SC participated in sample collection and bacterial isolation. ZY analyzed the genome sequences. SZ, QY, YW, and QG did the PFGE detection. XO, SM, JH, DS, and BT participated in analysis of antimicrobial resistance. All authors contributed to the article and approved the submitted version.

## Funding

This research was supported by the National Natural Science Foundation of China (32072877), the Applied Basic Research Programs of Science and Technology Department of Sichuan Province (2019YJ0436), and the earmarked fund for China Agriculture Research System (CARS-42-17).

## Conflict of interest

The authors declare that the research was conducted in the absence of any commercial or financial relationships that could be construed as a potential conflict of interest.

## Publisher’s note

All claims expressed in this article are solely those of the authors and do not necessarily represent those of their affiliated organizations, or those of the publisher, the editors and the reviewers. Any product that may be evaluated in this article, or claim that may be made by its manufacturer, is not guaranteed or endorsed by the publisher.
