# Supplementary material for: Phenotypic and genotypic characterization of antimicrobial resistance profiles in Salmonella isolated from waterfowl in 2002–2005 and 2018–2020 in Sichuan, China
Source: Front Microbiol. 2022 Oct 6;13:987613. doi: 10.3389/fmicb.2022.987613 (PMC9582774; doi:10.3389/fmicb.2022.987613)
Supplement: Supplementary file 1 [file Data_Sheet_1.PDF]

Supplementary Material

Supplementary Figures and Tables

Supplementary Figures

Supplementary Figure S1 | Dendrogram of the PFGE pulsotypes for the 126 *Salmonella* isolates (page 2). The orange line indicates the 60% cutoff value and the blue line indicates the 80% cutoff value. The “key” column represents the different isolates; RCAD, Research Center of Avian Diseases.

Supplementary Tables

Supplementary Table 1 | Comparison of average nucleotide identity of 8 isolates with similar band characteristics. The ANI levels between isolates with different serotypes were always lower than 99.0%, while the ANI levels of isolates with the same serotype were always larger than 99.0% regardless of their PFGE band distributions.

Isolate	Serotype	RCAD-S-014	RCAD-S-015	RCAD-S-016	RCAD-S-023	RCAD-S-024	RCAD-S-045
**RCAD-S-014**	**Montevideo**						
**RCAD-S-015**	**Potsdam**	**98.34%**					
**RCAD-S-016**	**Montevideo**	**99.99%**	**98.30%**				
**RCAD-S-020**	**Cerro**	**98.27%**	**98.56%**	**98.33%**	**99.91%**	**99.40%**	
**RCAD-S-023**	**Cerro**	**98.00%**	**98.10%**	**97.99%**		**99.79%**	
**RCAD-S-040**	**Enteritidis**	**98.44%**	**98.91%**	**98.47%**	**98.27%**	**98.36%**	**99.99%**

Supplementary Figure S1

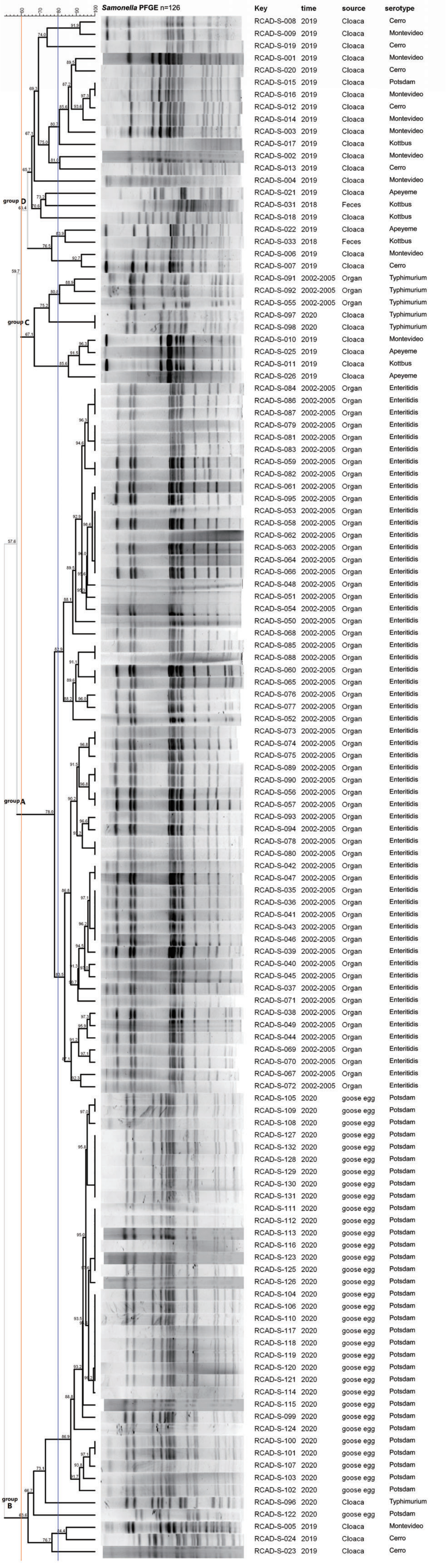

**Supplementary Table 2 | ** Inhibition zone sizes (mm) of the 126 *Salmonella* isolates against antimicrobial agents.

Isolate	Serotype	Time	MY 2ug I:15-20	RD 5ug I:17-19	SUL 100ug I:13-16	E 5ug I:14-22	CIP 5ug I:21-30	GEN 10ug I:13-15	EFT 30ug I:18-20	TET 30ug I:12-14	AMP 10ug I:14-16	STR 10ug I:12-14	ATM 30ug I:18-20	NAL 30ug I:14-18	IPM 10ug I:20-22	AML 25ug I:14-17	SXT 25ug I:11-15	CHL 30ug I:13-17	FEP 30ug R<19	W 5ug I:11-15	FFC 30ug I:15-18	PB 300ug I:8-11
RCAD-S-001	Montevideo	2018-2020	6	10	16	16	10	18	22	17	19	12	24	6	27	22	25	22	26	23	29	13
RCAD-S-002	Montevideo	2018-2020	6	10	15	6	16	19	19	16	6	14	22	6	26	20	23	21	25	21	29	14
RCAD-S-003	Montevideo	2018-2020	6	10	18	10	19	17	15	18	21	15	19	6	29	18	20	23	27	20	26	14
RCAD-S-004	Montevideo	2018-2020	6	10	14	6	18	18	15	16	19	14	6	6	26	20	23	22	26	20	25	13
RCAD-S-005	Montevideo	2018-2020	6	10	17	6	14	18	20	17	19	14	24	6	24	26	24	19	26	24	22	14
RCAD-S-006	Montevideo	2018-2020	6	8	6	16	20	19	16	6	7	14	22	19	21	20	23	21	25	19	25	14
RCAD-S-007	Cerro	2018-2020	6	9	6	6	17	20	16	15	7	12	18	17	22	18	6	21	22	6	25	14
RCAD-S-008	Cerro	2018-2020	6	6	6	14	22	10	6	18	10	14	6	6	25	6	6	25	24	6	25	14
RCAD-S-009	Montevideo	2018-2020	6	10	18	14	19	19	15	18	20	12	24	6	30	24	24	21	30	23	29	14
RCAD-S-010	Montevideo	2018-2020	7	6	6	16	6	12	17	15	7	7	22	6	23	21	22	17	25	20	24	13
RCAD-S-011	Kottbus	2018-2020	6	12	6	16	22	6	19	15	18	12	20	18	25	20	21	21	23	24	24	14
RCAD-S-012	Cerro	2018-2020	6	6	6	12	6	8	19	6	15	6	25	6	24	26	6	6	23	6	6	14
RCAD-S-013	Cerro	2018-2020	6	10	6	6	6	12	16	6	6	12	24	6	24	24	25	6	24	23	22	17
RCAD-S-014	Montevideo	2018-2020	6	11	15	6	18	6	19	16	20	14	25	6	25	22	22	22	29	24	29	14
RCAD-S-015	Potsdam	2018-2020	6	10	19	16	24	10	21	18	20	9	29	15	26	22	6	25	24	26	29	14
RCAD-S-016	Montevideo	2018-2020	6	6	6	10	6	11	10	6	6	10	18	6	21	6	23	10	19	20	6	12
RCAD-S-017	Kottbus	2018-2020	6	10	6	15	22	8	13	16	18	12	6	19	24	18	20	22	25	21	26	14
RCAD-S-018	Kottbus	2018-2020	6	10	6	10	30	11	6	18	19	11	6	20	27	19	20	24	25	19	25	12
RCAD-S-019	Cerro	2018-2020	6	10	6	6	24	13	15	18	18	15	16	6	27	22	24	22	25	24	25	15
RCAD-S-020	Cerro	2018-2020	6	9	6	6	20	10	13	14	11	11	19	6	24	20	20	20	20	22	24	15
RCAD-S-021	Apeyeme	2018-2020	6	10	6	10	12	6	16	12	14	9	24	17	14	22	23	18	20	22	26	15
RCAD-S-022	Apeyeme	2018-2020	6	8	6	14	6	6	18	13	15	8	22	18	14	20	24	21	21	23	27	13
RCAD-S-023	Cerro	2018-2020	6	8	6	15	6	6	6	13	6	11	19	18	22	6	21	20	20	24	26	14
RCAD-S-024	Cerro	2018-2020	6	10	6	16	10	10	16	6	6	12	18	19	20	8	20	22	22	22	28	16
RCAD-S-025	Apeyeme	2018-2020	6	9	6	10	14	14	16	6	15	12	6	19	24	19	18	18	20	20	24	14
RCAD-S-026	Apeyeme	2018-2020	6	6	6	6	11	6	12	12	15	10	18	17	22	18	18	19	18	18	24	11
RCAD-S-031	Kottbus	2018-2020	6	6	6	6	6	6	6	6	6	6	20	6	16	6	6	6	6	6	6	14
RCAD-S-033	Kottbus	2018-2020	6	10	6	6	6	20	24	16	16	13	30	14	22	29	28	9	21	30	26	16
RCAD-S-035	Enteritidis	2002-2005	6	12	6	6	6	6	16	6	10	12	22	14	19	18	17	16	19	20	25	15
RCAD-S-036	Enteritidis	2002-2005	6	10	6	6	6	6	16	6	12	16	21	17	23	21	20	18	19	21	24	15
RCAD-S-037	Enteritidis	2002-2005	6	10	6	6	6	6	19	6	12	15	15	17	23	20	22	20	19	24	27	12
RCAD-S-038	Enteritidis	2002-2005	6	10	6	10	17	6	6	13	12	15	15	16	22	20	18	20	20	21	27	14
RCAD-S-039	Enteritidis	2002-2005	6	10	6	6	6	6	6	15	12	6	16	16	21	19	18	19	18	18	26	14
RCAD-S-040	Enteritidis	2002-2005	6	9	6	6	6	6	18	14	15	14	6	17	25	20	19	20	23	21	27	12
RCAD-S-041	Enteritidis	2002-2005	6	10	6	10	12	6	6	6	17	12	6	18	23	19	19	20	23	18	26	13
RCAD-S-042	Enteritidis	2002-2005	6	10	6	15	6	6	17	6	19	6	20	19	25	20	21	20	23	20	25	13
RCAD-S-043	Enteritidis	2002-2005	6	11	6	12	6	6	6	15	14	17	10	18	24	20	6	20	21	18	26	14
RCAD-S-044	Enteritidis	2002-2005	6	11	6	14	15	6	6	6	13	10	6	18	25	20	20	20	21	18	28	14
RCAD-S-045	Enteritidis	2002-2005	6	10	6	12	10	6	18	6	6	6	21	6	27	6	18	6	21	22	28	13
RCAD-S-046	Enteritidis	2002-2005	6	12	6	12	14	6	6	6	13	16	6	19	24	19	18	20	19	19	25	13
RCAD-S-047	Enteritidis	2002-2005	6	11	6	12	10	20	15	6	14	16	20	18	22	22	21	17	18	21	26	13
RCAD-S-048	Enteritidis	2002-2005	6	9	6	6	6	6	10	6	11	16	15	16	20	20	20	13	19	21	25	12
RCAD-S-049	Enteritidis	2002-2005	6	10	6	6	6	6	15	13	13	15	19	18	23	21	19	20	19	20	24	11
RCAD-S-050	Enteritidis	2002-2005	6	12	6	12	10	6	6	6	12	15	18	17	22	21	19	20	21	20	24	16
RCAD-S-051	Enteritidis	2002-2005	6	10	6	6	6	19	24	6	17	16	32	19	22	26	27	22	22	24	29	14
RCAD-S-052	Enteritidis	2002-2005	6	8	6	6	6	6	22	14	12	15	30	17	21	26	24	21	19	23	27	15
RCAD-S-053	Enteritidis	2002-2005	6	10	6	6	6	6	25	6	13	15	31	17	19	23	23	19	21	23	26	17
RCAD-S-054	Enteritidis	2002-2005	6	12	6	6	6	6	22	6	13	15	32	17	20	26	27	20	18	24	28	15
RCAD-S-055	Typhimurium	2002-2005	6	8	6	6	6	6	23	14	15	15	27	19	26	26	26	21	25	23	25	15
RCAD-S-056	Enteritidis	2002-2005	6	10	6	6	6	6	23	6	17	17	30	18	25	25	28	17	22	24	28	15
RCAD-S-057	Enteritidis	2002-2005	6	10	6	6	6	6	24	6	6	6	33	6	24	6	20	6	20	24	30	16
RCAD-S-058	Enteritidis	2002-2005	6	6	6	6	15	6	25	6	15	15	30	16	21	23	25	20	22	23	26	14
RCAD-S-059	Enteritidis	2002-2005	6	12	6	6	6	6	20	6	13	16	28	17	23	22	24	20	22	23	26	15
RCAD-S-060	Enteritidis	2002-2005	6	6	6	6	6	6	19	14	16	15	30	17	20	24	23	18	22	22	25	14
RCAD-S-061	Enteritidis	2002-2005	6	8	6	6	6	19	24	6	13	14	33	18	23	26	26	20	20	23	24	14
RCAD-S-062	Enteritidis	2002-2005	6	8	6	6	6	20	24	6	14	15	29	17	23	25	24	19	22	22	23	15
RCAD-S-063	Enteritidis	2002-2005	6	9	6	6	14	6	22	6	15	17	30	16	22	25	25	19	18	24	25	15
RCAD-S-064	Enteritidis	2002-2005	6	9	6	6	6	6	21	6	11	6	29	19	24	24	24	18	20	22	24	15
RCAD-S-065	Enteritidis	2002-2005	6	8	6	6	6	6	22	15	14	6	28	17	22	23	23	19	20	24	22	18
RCAD-S-066	Enteritidis	2002-2005	6	8	6	6	6	6	21	6	14	17	28	18	20	20	20	23	20	20	25	15
RCAD-S-067	Enteritidis	2002-2005	6	12	6	6	22	6	22	6	14	17	29	17	22	24	22	17	18	22	29	14
RCAD-S-068	Enteritidis	2002-2005	6	8	6	6	6	6	21	6	14	17	29	16	21	24	22	19	20	22	25	15
RCAD-S-069	Enteritidis	2002-2005	6	6	6	6	6	6	20	15	15	6	25	18	24	23	23	22	22	22	23	14
RCAD-S-070	Enteritidis	2002-2005	6	6	6	6	6	6	20	15	17	16	28	19	20	23	22	21	25	20	23	14
RCAD-S-071	Enteritidis	2002-2005	6	8	6	6	25	6	24	6	16	19	33	18	25	26	24	20	24	22	28	15
RCAD-S-072	Enteritidis	2002-2005	6	8	6	6	6	6	21	15	20	17	29	19	22	25	22	22	25	23	28	14
RCAD-S-073	Enteritidis	2002-2005	6	6	6	6	19	10	22	6	17	18	28	19	15	24	22	23	25	20	24	13
RCAD-S-074	Enteritidis	2002-2005	6	6	6	6	17	12	20	6	6	6	30	6	16	6	18	6	25	24	27	13
RCAD-S-075	Enteritidis	2002-2005	6	6	6	6	20	10	19	6	19	17	25	20	17	24	24	23	27	20	25	15
RCAD-S-076	Enteritidis	2002-2005	6	8	6	6	6	6	22	6	18	17	30	20	17	25	24	24	26	23	27	14
RCAD-S-077	Enteritidis	2002-2005	6	8	6	6	17	6	20	16	18	16	29	20	16	24	23	21	26	20	28	14
RCAD-S-078	Enteritidis	2002-2005	6	6	6	6	17	8	22	6	16	19	29	20	19	23	22	22	24	21	24	14
RCAD-S-079	Enteritidis	2002-2005	6	8	6	6	17	6	23	6	17	17	25	21	18	22	21	22	25	22	26	14
RCAD-S-080	Enteritidis	2002-2005	6	6	6	6	19	19	20	6	15	16	23	20	25	20	20	20	21	20	25	14
RCAD-S-081	Enteritidis	2002-2005	6	8	6	6	19	18	20	6	16	18	28	18	24	25	25	20	23	25	27	15
RCAD-S-082	Enteritidis	2002-2005	6	6	6	6	20	19	24	6	18	18	30	19	25	24	24	22	24	25	28	16
RCAD-S-083	Enteritidis	2002-2005	6	8	6	6	19	20	24	6	18	17	32	20	24	25	26	21	25	25	30	14
RCAD-S-084	Enteritidis	2002-2005	6	10	6	6	22	20	19	6	18	19	30	20	26	24	23	23	24	22	25	14
RCAD-S-085	Enteritidis	2002-2005	6	8	6	6	21	19	22	17	18	17	28	19	26	26	25	22	22	24	25	14
RCAD-S-086	Enteritidis	2002-2005	6	6	6	6	23	21	19	6	17	18	28	20	25	24	23	22	21	22	25	15
RCAD-S-087	Enteritidis	2002-2005	6	6	6	6	21	18	20	6	17	19	28	20	26	23	24	20	24	22	25	13
RCAD-S-088	Enteritidis	2002-2005	6	8	6	6	21	17	20	17	19	17	30	20	26	24	26	21	25	23	26	13
RCAD-S-089	Enteritidis	2002-2005	6	6	6	6	22	19	20	6	16	15	28	20	28	25	25	20	24	23	25	14
RCAD-S-090	Enteritidis	2002-2005	6	6	6	6	19	6	18	6	19	17	22	21	25	20	20	18	24	20	24	14
RCAD-S-091	Typhimurium	2002-2005	6	8	6	6	19	20	20	16	19	13	27	20	20	26	20	23	25	21	25	15
RCAD-S-092	Typhimurium	2002-2005	6	8	6	6	20	22	19	6	20	17	22	22	27	20	24	25	28	21	25	14
RCAD-S-093	Enteritidis	2002-2005	6	6	6	6	20	6	6	6	21	19	6	22	20	23	20	24	27	20	27	13
RCAD-S-094	Enteritidis	2002-2005	6	8	6	6	20	20	19	6	20	17	24	21	20	24	26	22	27	22	25	9
RCAD-S-095	Enteritidis	2002-2005	6	6	6	6	18	17	18	6	6	6	25	6	25	6	9	6	30	19	26	13
RCAD-S-096	Typhimurium	2018-2020	6	10	6	12	17	6	18	17	17	11	20	18	24	22	24	24	26	24	29	10
RCAD-S-097	Typhimurium	2018-2020	6	6	6	6	16	6	16	17	18	6	26	19	25	20	20	25	27	21	17	12
RCAD-S-098	Typhimurium	2018-2020	6	6	6	6	6	6	16	18	18	13	26	19	26	21	22	25	27	20	23	7
RCAD-S-099	Potsdam	2018-2020	6	6	6	6	17	6	17	20	19	14	20	18	26	20	23	24	25	24	23	8
RCAD-S-100	Potsdam	2018-2020	6	6	6	6	6	6	19	18	20	13	29	18	28	27	25	23	28	20	15	6
RCAD-S-101	Potsdam	2018-2020	6	8	6	6	6	8	22	18	18	12	30	20	27	24	26	23	26	23	30	14
RCAD-S-102	Potsdam	2018-2020	6	8	6	6	6	6	20	18	20	12	30	19	26	24	25	24	25	23	25	15
RCAD-S-103	Potsdam	2018-2020	6	10	6	6	6	6	20	18	20	14	32	19	25	28	29	24	26	26	29	17
RCAD-S-104	Potsdam	2018-2020	6	8	6	6	6	6	20	19	20	12	31	20	25	24	27	25	27	25	28	15
RCAD-S-105	Potsdam	2018-2020	6	10	6	6	17	6	6	16	22	13	19	18	26	20	22	24	29	20	25	13
RCAD-S-106	Potsdam	2018-2020	6	8	6	6	19	6	20	18	21	14	24	19	26	20	21	25	28	20	26	13
RCAD-S-107	Potsdam	2018-2020	6	10	6	6	22	6	15	17	19	12	6	18	24	22	6	20	24	16	26	12
RCAD-S-108	Potsdam	2018-2020	6	8	6	6	18	6	6	18	19	6	6	20	25	19	20	20	26	20	20	14
RCAD-S-109	Potsdam	2018-2020	6	12	6	6	17	6	6	20	22	14	19	19	25	20	20	24	26	20	26	13
RCAD-S-110	Potsdam	2018-2020	6	7	6	6	18	6	15	19	17	6	20	16	23	21	20	21	24	17	22	12
RCAD-S-111	Potsdam	2018-2020	6	8	6	6	20	6	14	18	14	13	17	17	22	19	21	22	24	19	26	12
RCAD-S-112	Potsdam	2018-2020	6	7	6	6	16	6	14	19	19	14	23	18	24	21	23	22	23	21	22	13
RCAD-S-113	Potsdam	2018-2020	6	12	6	6	20	6	14	19	19	14	20	18	24	21	23	23	26	21	26	12
RCAD-S-114	Potsdam	2018-2020	6	10	6	6	16	6	14	17	20	13	20	18	24	22	22	22	25	22	23	13
RCAD-S-115	Potsdam	2018-2020	6	10	6	6	18	6	15	17	21	6	19	17	26	20	24	22	25	24	26	13
RCAD-S-116	Potsdam	2018-2020	6	10	6	6	19	11	16	17	19	14	26	19	24	24	22	22	27	22	25	14
RCAD-S-117	Potsdam	2018-2020	6	10	6	6	21	10	16	18	23	14	19	18	26	22	23	23	29	24	25	13
RCAD-S-118	Potsdam	2018-2020	6	13	6	6	20	11	15	18	19	13	19	18	26	23	20	22	27	20	23	14
RCAD-S-119	Potsdam	2018-2020	6	12	6	6	20	11	16	20	15	13	20	18	25	21	23	22	24	20	22	14
RCAD-S-120	Potsdam	2018-2020	6	12	6	6	25	9	15	18	20	14	20	18	27	24	21	23	29	21	26	14
RCAD-S-121	Potsdam	2018-2020	6	10	6	6	20	10	6	17	19	14	21	18	27	21	23	24	28	24	26	12
RCAD-S-122	Potsdam	2018-2020	6	6	6	6	22	9	15	18	20	14	22	17	25	23	22	24	27	21	28	13
RCAD-S-123	Potsdam	2018-2020	6	8	6	6	20	9	17	18	19	15	24	19	25	21	21	21	26	21	23	12
RCAD-S-124	Potsdam	2018-2020	6	7	6	6	22	8	19	19	17	13	25	18	26	24	25	23	27	22	25	15
RCAD-S-125	Potsdam	2018-2020	6	10	6	6	24	6	17	18	19	14	22	21	28	23	24	24	28	20	28	12
RCAD-S-126	Potsdam	2018-2020	6	10	6	6	20	6	16	18	20	14	22	19	26	22	25	23	27	23	25	13
RCAD-S-127	Potsdam	2018-2020	6	6	6	6	20	6	6	19	21	15	6	20	27	6	6	23	30	6	6	12
RCAD-S-128	Potsdam	2018-2020	6	8	6	6	22	11	6	16	22	15	6	20	29	20	20	24	29	21	24	13
RCAD-S-129	Potsdam	2018-2020	6	7	6	6	20	10	18	17	22	14	24	20	27	21	24	24	29	24	25	13
RCAD-S-130	Potsdam	2018-2020	6	7	6	6	24	18	16	17	21	13	20	20	25	21	23	23	25	22	25	13
RCAD-S-131	Potsdam	2018-2020	6	10	6	6	22	6	20	15	19	12	22	21	27	22	23	22	24	23	23	13
RCAD-S-132	Potsdam	2018-2020	6	8	6	6	25	19	20	18	18	13	20	21	25	21	25	22	22	23	20	14
SUM			126	126	118	113	102	95	59	52	31	26	21	19	12	10	9	9	7	5	4	2
R+ %			100.0	100.0	93.7	89.7	81.0	75.4	46.8	41.3	24.6	20.6	16.7	15.1	9.5	7.9	7.1	7.1	5.6	4.0	3.2	1.6
2002-2005			61	61	61	59	53	43	14	45	20	10	10	4	9	4	2	4	5	0	0	0
R+ %			100.0	100.0	100.0	96.7	86.9	70.5	23.0	73.8	32.8	16.4	16.4	6.6	14.8	6.6	3.3	6.6	8.2	0.0	0.0	0.0
2018-2020			65	65	57	54	49	52	45	7	11	16	11	15	3	6	7	5	2	5	4	2
R+ %			100.0	100.0	87.7	83.1	75.4	80.0	69.2	10.8	16.9	24.6	16.9	23.1	4.6	9.2	10.8	7.7	3.1	7.7	6.2	3.1

Red, resistant (R); yellow, susceptible, increased exposure (I); and grey, susceptible, standard dosing regimen (S). SUL, sulfadiazine; CIP, ciprofloxacin; GEN, gentamycin; EFT, ceftiofur; TET, tetracycline; AMP, ampicillin; STR, streptomycin; ATM, aztreonam; NAL, nalidixic acid; IPM, imipenem; SXT, trimethoprim/sulfamethoxazole; AML, amoxicillin; CHL, chloramphenicol; FEP, cefepime; W, trimethoprim; FFC, florfenicol; and PB, polymyxin B.

Supplementary Table 3 | Full version of AMR genes for the 49 isolates.

Isolate	AMR Gene																																											
	mdsA	*adeF*	*golS*	*sdiA*	*acrA/B*	*marA/R*	*baeR/S*	*rsmA*	*crp*	*H-NS*	*mdtK*	*mdfA*	*kpnE/F*	*emrB/R*	*soxS/R*	*msbA*	*glpT*	*uhpT*	*EF-Tu*	*kdpE*	*aac(6')-Iy*	*aac(6')-Iaa*	*aac(3)-IId*	*aph(3')-Ia*	*aph(3'')-Ib*	*aph(6)-Id*	*ampC1/H*	*pbp3*	*bla_{TEM-1}*	*$bla_{TEM-116}$*	*catII*	*dfrA27*	*sul1*	*sul2*	*pmrF*	*bacA*	*mcr-1.1*	*gyrA*	*gyrB*	*qnrB6*	*tet* (A)	*tet* (B)	*tetR*	*arr-3*	*qacEdelta1*
RCAD-S-001		—	—	—	—	—	—	—	—	—	—	—	—	—	—	—	—	—	—	—	—						—	—							—	—		S83F							
RCAD-S-002		—	—	—	—	—	—	—	—	—	—	—	—	—	—	—	—	—	—	—	—						—	—							—	—		S83F							
RCAD-S-003		—	—	—	—	—	—	—	—	—	—	—	—	—	—	—	—	—	—	—	—						—	—							—	—		S83F							
RCAD-S-004		—	—	—	—	—	—	—	—	—	—	—	—	—	—	—	—	—	—	—	—						—	—							—	—		S83F							
RCAD-S-005		—	—	—	—	—	—	—	—	—	—	—	—	—	—	—	—	—	—	—	—						—	—		—					—	—		S83F							
RCAD-S-008	—	—	—	—	—	—	—	—	—	—	—	—	—	—	—	—	—	—	—	—	—		—				—	—				—	—		—	—		D87N		—				—	—
RCAD-S-009		—	—	—	—	—	—	—	—	—	—	—	—	—	—	—	—	—	—	—	—						—	—							—	—		S83F							
RCAD-S-010		—	—	—	—	—	—	—	—	—	—	—	—	—	—	—	—	—	—	—	—						—	—							—	—		S83F							
RCAD-S-011		—	—	—	—	—	—	—	—	—	—	—	—	—	—	—	—	—	—	—	—						—	—							—	—									
RCAD-S-013	—	—	—	—	—	—	—	—	—	—	—	—	—	—	—	—	—	—	—	—	—						—	—							—	—		D87N							
RCAD-S-014		—	—	—	—	—	—	—	—	—	—	—	—	—	—	—	—	—	—	—	—						—	—							—	—		S83F							
RCAD-S-015		—	—	—	—	—	—	—	—	—	—	—	—	—	—	—	—	—	—	—	—						—	—							—	—	—		E466D						
RCAD-S-017		—	—	—	—	—	—	—	—	—	—	—	—	—	—	—	—	—	—	—	—						—	—							—	—									
RCAD-S-018		—	—	—	—	—	—	—	—	—	—	—	—	—	—	—	—	—	—	—	—						—	—							—	—									
RCAD-S-019	—	—	—	—	—	—	—	—	—	—	—	—	—	—	—	—	—	—	—	—	—						—	—							—	—		D87N							
RCAD-S-020		—	—	—	—	—	—	—	—	—	—	—	—	—	—	—	—	—	—	—	—						—	—							—	—		D87N							
RCAD-S-021	—	—	—	—	—	—	—	—	—	—	—	—	—	—	—	—	—	—	—	—	—						—	—							—	—									
RCAD-S-022	—	—	—	—	—	—	—	—	—	—	—	—	—	—	—	—	—	—	—	—	—						—	—							—	—									
RCAD-S-025	—	—	—	—	—	—	—	—	—	—	—	—	—	—	—	—	—	—	—	—	—						—	—							—	—									
RCAD-S-033		—	—	—	—	—	—	—	—	—	—	—	—	—	—	—	—	—	—	—	—						—	—							—	—									
RCAD-S-035		—	—	—	—	—	—	—	—	—	—	—	—	—	—	—	—	—	—	—	—						—	—							—	—						—	—		
RCAD-S-039		—	—	—	—	—	—	—	—	—	—	—	—	—	—	—	—	—	—	—	—						—	—							—	—						—	—		
RCAD-S-040		—	—	—	—	—	—	—	—	—	—	—	—	—	—	—	—	—	—	—	—						—	—							—	—									
RCAD-S-042		—	—	—	—	—	—	—	—	—	—	—	—	—	—	—	—	—	—	—	—						—	—							—	—						—	—		
RCAD-S-044		—	—	—	—	—	—	—	—	—	—	—	—	—	—	—	—	—	—	—	—						—	—							—	—						—	—		
RCAD-S-045		—	—	—	—	—	—	—	—	—	—	—	—	—	—	—	—	—	—	—	—				—	—	—	—	—		—			—	—	—		D87G			—				
RCAD-S-050		—	—	—	—	—	—	—	—	—	—	—	—	—	—	—	—	—	—	—	—						—	—							—	—						—	—		
RCAD-S-051		—	—	—	—	—	—	—	—	—	—	—	—	—	—	—	—	—	—	—	—						—	—							—	—						—	—		
RCAD-S-055	—		—	—	—	—	—	—	—	—	—		—	—	—	—	—	—	—	—		—					—	—							—	—									
RCAD-S-057		—	—	—	—	—	—	—	—	—	—	—	—	—	—	—	—	—	—	—	—				—	—	—	—	—		—			—	—	—		D87G			—				
RCAD-S-058		—	—	—	—	—	—	—	—	—	—	—	—	—	—	—	—	—	—	—	—						—	—							—	—						—	—		
RCAD-S-060		—	—	—	—	—	—	—	—	—	—	—	—	—	—	—	—	—	—	—	—						—	—							—	—									
RCAD-S-065		—	—	—	—	—	—	—	—	—	—	—	—	—	—	—	—	—	—	—	—						—	—							—	—									
RCAD-S-067		—	—	—	—	—	—	—	—	—	—	—	—	—	—	—	—	—	—	—	—						—	—							—	—						—	—		
RCAD-S-071		—	—	—	—	—	—	—	—	—	—	—	—	—	—	—	—	—	—	—	—						—	—							—	—						—	—		
RCAD-S-074		—	—	—	—	—	—	—	—	—	—	—	—	—	—	—	—	—	—	—	—				—	—	—	—	—		—			—	—	—		D87G			—				
RCAD-S-085		—	—	—	—	—	—	—	—	—	—	—	—	—	—	—	—	—	—	—	—						—	—							—	—									
RCAD-S-089		—	—	—	—	—	—	—	—	—	—	—	—	—	—	—	—	—	—	—	—						—	—							—	—						—	—		
RCAD-S-091	—		—	—	—	—	—	—	—	—	—		—	—	—	—	—	—	—	—		—					—	—							—	—									
RCAD-S-095		—	—	—	—	—	—	—	—	—	—	—	—	—	—	—	—	—	—	—	—				—	—	—	—	—		—			—	—	—		D87G			—				
RCAD-S-096	—		—	—	—	—	—	—	—	—	—	—	—	—	—	—	—	—	—	—		—		—			—	—							—	—									
RCAD-S-097	—	—	—	—	—	—	—	—	—	—	—		—	—	—	—	—	—	—	—		—					—	—							—	—									
RCAD-S-100		—	—	—	—	—	—	—	—	—	—	—	—	—	—	—	—	—	—	—	—						—	—							—	—									
RCAD-S-107		—	—	—	—	—	—	—	—	—	—	—	—	—	—	—	—	—	—	—	—			—			—	—							—	—									
RCAD-S-108		—	—	—	—	—	—	—	—	—	—	—	—	—	—	—	—	—	—	—	—						—	—							—	—									
RCAD-S-113		—	—	—	—	—	—	—	—	—	—	—	—	—	—	—	—	—	—	—	—						—	—							—	—									
RCAD-S-116		—	—	—	—	—	—	—	—	—	—	—	—	—	—	—	—	—	—	—	—						—	—							—	—									
RCAD-S-122		—	—	—	—	—	—	—	—	—	—	—	—	—	—	—	—	—	—	—	—						—	—							—	—									
RCAD-S-129		—	—	—	—	—	—	—	—	—	—	—	—	—	—	—	—	—	—	—	—			—			—	—							—	—									

—, Hit resistance genes. For the *gyrA* and *gyrB* genes, mutation sites are shown.
